# Protective effect of *Schistosoma japonicum* eggs on TNBS-induced colitis is associated with regulating Treg/Th17 balance and reprogramming glycolipid metabolism in mice

**DOI:** 10.3389/fcimb.2022.1028899

**Published:** 2022-10-11

**Authors:** Xiao Hou, Feifan Zhu, Wenwen Zheng, Muziazia Lupemba Jacques, Jin Huang, Fei Guan, Jiahui Lei

**Affiliations:** ^1^ Department of Clinical Laboratory, The General Hospital of Central Theater Command, The People's Liberation Army, Wuhan, China; ^2^ Department of Pathogen Biology, School of Basic Medicine, Tongji Medical College, Huazhong University of Science and Technology, Wuhan, China; ^3^ Department of Parasitology, Kinshasa Institute of Medical, Kinshasa, Democratic Republic of the Congo; ^4^ Department of Clinical Laboratory, Wuhan Pu’ai Hospital, Wuhan, China

**Keywords:** *Schistosoma japonicum* eggs, TNBS, colitis, Treg/Th17 balance, glycolipid metabolism

## Abstract

Inflammatory bowel diseases (IBDs) have been classified as modern refractory diseases. However, safe, well-tolerated, and effective treatments for IBDs are still lacking. Therefore, there is an urgent need to develop novel therapeutic targets with fewer undesirable adverse reactions. A growing body of research has shown that infection with live helminths or exposure to defined helminth-derived components can downregulate pathogenic inflammation due to their immunoregulatory ability. Here we were to explore the protective role of *Schistosoma japonicum* eggs on murine experimental colitis caused by trinitrobenzene sulfonic acid (TNBS) and the underlying mechanism. Frequencies of splenic Treg and Th17 cells were detected by flow cytometry. Protein and mRNA expressions of Foxp3 and RORγt were investigated by Western Blot and quantitative real-time polymerase chain reaction (qPCR), respectively. Concentrations of transforming growth factor-beta1 (TGF-β1), interleukin-10 (IL-10) and IL-17A were assessed with ELISA. Expression levels of genes related to glycolipid metabolism were measured with qPCR. The results showed that pre-exposure to *S. japonicum* eggs contributed to the relief of colitis in the TNBS model, evidenced by improved body weight loss, reversing spleen enlargement and colon shortening, and decreased histology scores. Compared with the TNBS group, the TNBS+Egg group had increased Treg immune response, accompanied by decreased Th17 immune response, leading to the reconstruction of Treg/Th17 balance. In addition, a ratio of Treg/Th17 was correlated negatively with the histological scores in the experiment groups. Furthermore, the regulation of Treg/Th17 balance by *S. japonicum* eggs was associated with inhibiting the glycolysis pathway and lipogenesis, along with promoting fatty acid oxidation in the TNBS+Egg group. These data indicate that *S. japonicum* eggs have a protective effect against TNBS-induced colitis, which is related to restoring Treg/Th17 balance and regulating glucose and lipid metabolism.

## Introduction

Inflammatory bowel diseases (IBDs) constitute a group of idiopathic inflammatory bowel disorders prominently characterized by intestinal immune system dysfunction and metabolic disorders (Chang, 2020). IBDs have been classified as modern refractory diseases by the WHO, with Crohn’s disease (CD) and ulcerative colitis (UC) as two main forms (Hodson, 2016; Na and Moon, 2019). IBDs are quite common in the West, with roughly 200-300 cases per million people. And the incidence of IBDs in the East has soared over the past decade to become a global disease (Mak et al., 2020). However, safe, well-tolerated, and effective treatments for IBDs are still lacking. Therefore, there is an urgent need to develop novel therapeutic targets with fewer undesirable adverse reactions (Na and Moon, 2019).

The pursuit of cleanliness and the subsequent loss of intestinal worms have played an important role in IBDs, although the reasons for the growing cases are not fully understood. And immunological factors including dysregulation of the immune response are associated with IBDs pathogenesis (Savage, 2016). A growing body of research in mouse models has shown that infection with live helminths or exposure to defined helminth-derived components can downregulate pathogenic inflammation due to their immunoregulatory ability (Shi et al., 2022). *Schistosoma* and its active proteins show a strong ability for immune regulation in a variety of immunological disorders (Mu et al., 2021). It has been shown that *Schistosoma mansoni* eggs can prevent the development of experimental colitis induced by 2, 4, 6-trinitrobenzene sulfonic acid (TNBS) (Elliott et al., 2003). Results from our group and others have shown that *Schistosoma japonicum* eggs also prevent the development of experimental colitis induced by TNBS (Mo et al., 2007; Zhao et al., 2009). However, the underlying immune mechanism of schistosome eggs down-regulating the pathogenic response of experimental colitis needs to be further explored. Therefore, a greater understanding of the protective roles of helminths on intestinal inflammation is essential for the development of effective and safe treatments for IBDs.

Growing research works have reported that inflammatory diseases are associated with regulatory T (Treg) and T helper 17 (Th17) imbalance (Noack and Miossec, 2014). The development and progression of IBDs are associated with the delicate balance between anti- and pro-inflammatory cytokines (Neurath, 2014). Disruption of Treg/Th17 is considered to be an essential cause of IBDs, among them Treg cells suppress tissue inflammation whereas Th17 cells represent a pro-inflammatory subset (Yan et al., 2020). Soluble egg antigen (SEA) of *S. mansoni* can upregulate Treg cells in a diabetes model (Zaccone et al., 2009). Soluble proteins of *S. mansoni* increase mRNA expression of transforming growth factor-beta (TGF-β) and interleukin-10 (IL-10) while suppressing expression of interleukin-17 (IL-17) in murine TNBS-induced colitis (Ruyssers et al., 2009). Therefore, the protective effect of *Schistosoma* eggs on immune-mediated disorders is not only related to Treg, but also associated with Th17. Whether *S. japonicum* eggs prevent experimental colitis *via* regulating Treg/Th17 balance or not, has not yet been reported.

Recently, increasing studies indicate that metabolic disorders modulate the pathogenesis of IBDs by regulating immune responses (Aden et al., 2019; Lamb et al., 2022; Wang et al., 2022). Furthermore, glucose and lipid metabolism play important roles in T cells’ development and functions, even controlling the balance between Th17/Treg (Qin et al., 2022). It has been proved that both *S. japonicum* infection and SEA result in the reprogramming of glucose and lipid metabolism in mice (Xu et al., 2019; Cai et al., 2021; Chen et al., 2021). Therefore, the insights into the effects of egg treatment on glucose and lipid metabolic changes will shed light on the underlying mechanism in IBDs.

Based on the above findings, this study observed the effect of *S. japonicum* eggs on the disease development of experimental colitis induced by TNBS in a mouse model. Treg and Th17 immune responses were detected in the experimental groups. Furthermore, to determine the exact therapeutic mechanisms of egg treatment in the murine experimental colitis, glucose and lipid metabolism in T cells treated with SEA *in vitro* and in the colon from the mouse model were explored, too.

## Materials and methods

### Collection of *S. japonicum* eggs

*S. japonicum* eggs were harvested from livers of rabbits at days 46 post infection with 1500 cercariae as previously described (Mo et al., 2007). Livers of the infected rabbits were collected and cut into small pieces. Then the liver pieces were homogenized in phosphate-buffered saline (PBS) on ice, filtered, washed, and centrifuged at 12,000 rpm for 15min. And centrifuged eggs were suspended in PBS and stored in liquid nitrogen for further use.

### Preparation of SEA

SEA was prepared as previously described (Zhu et al., 2014). Briefly, purified eggs were suspended in pre-cooled PBS of 500 µL with 1 mM Phenylmethanesulfonyl fluoride (PMSF, Roche Diagnostics) and 2 μg/mL Leupeptin (Sigma), and then homogenized on ice with a homogenizer (VirTis Co.). The suspension was frozen and thawed several times and centrifuged at 12,000 rpm for 50 min at 4 °C. SEA in the supernatant was obtained after filtering through a 0.22 μm filter (Millipore Corporation). The protein concentration was determined with the BCA Protein Assay Kit (Beyotime Biotech, Beijing, China).

### Induction of murine experimental colitis and egg treatment

Eighteen female BALB/c mice, aged 6-8 weeks, were purchased from the Laboratory Animal Center at Tongji Medical College, China. All mice (five per cage) were maintained in a standard specific pathogen-free research animal facility with food and water provided *ad libitum*. Mice were randomly divided into three groups: the Control group, TNBS, and TNBS + Egg group. Mice from the TNBS + Egg group were intraperitoneally injected with 10,000 thaw-killed eggs in PBS (100 µL) on days 14 and 10 before TNBS administration, respectively, as previously described (Elliott et al., 2003). The control and TNBS groups were intraperitoneally injected with the same volume of PBS at the corresponding time. On day 0, TNBS (0.5 mg per mouse in 50% ethanol) was challenged in mice to induce colitis in the TNBS and TNBS + Egg groups. It is reported that mice are sacrificed between days 3 and 7 after intrarectal challenge with TNBS in models of acute colitis, because intestinal inflammation is usually strongest at this stage (Wirtz et al., 2017). Generally, mice were sacrificed and analyzed on the 5^th^ day after TNBS administration unless otherwise indicated.

### Assessment of activities, appetite, body weight, and spleen index

Activities and appetite of all mice were monitored during the experiment. Mice were weighed and recorded daily. The body weight change was determined by calculating the percentage of weight change relative to the corresponding body weight at day 0. The proportion of the spleen weight to its corresponding body weight was calculated and recorded as spleen index at the end of the experiment.

### Stimulation of CD4+ T cells with SEA *in vitro*

Spleen naïve CD4+ T cells (CD4^+^ CD25^−^ CD44^−^ CD62L^+^) were sorted from the TNBS mice as previously described (Bai et al., 2021). Naïve CD4+ T cells were then cultured (3×10^5^ cells/well) in 96-well plates coated with anti-CD3 mAb (5 μg/mL) and soluble anti-CD28 mAb (3 μg/mL) and kept in the presence of IL-2 (100 U/mL). All antibodies were purchased from eBioscience. SEA (10 µg/mL) was added and PBS was used as the control. Cells were cultured in RPMI- 1640 medium, 10% fetal calf serum (FCS), 2 mM L-glutamine, 100 U/mL of Penicillin/Streptomycin for 5 days.

### Evaluation of colitis inflammation

In addition to analysis of body weight, routine methods for the analysis of degree of intestinal inflammation in colitis include colon length, hematoxylin-eosin (HE) staining of colon tissues, and histopathological scoring (Wirtz et al., 2017). Thus, colons were collected and their extents were recorded. The proximal 1.0 cm of the colonic segment was fixed in 4% formaldehyde and embedded in paraffin. Morphometric analysis was performed on the HE-stained section. The extent of damage and colonic inflammation was graded from 0 to 4 in a blinded fashion by two investigators, according to the histopathological grading system of Neurath et al. (Neurath et al., 1995). The scoring system described previously was 0 = no inflammation, 1= low-level of leukocyte infiltration, 2 = intermediate- level of leukocyte infiltration, 3 = high-level of leukocyte infiltration with vascular density and wall thickening, 4 = transmural leukocyte infiltration, loss of goblet cells, high vascular density, and wall thickening.

### Flow cytometric analysis

Flow cytometry analyses were performed to assess the proportion of Treg and Th17 cells in spleens from all groups. Briefly, spleen lymphocytes were purified using lympholyte M (Cedarlane, Ontario, Canada) and washed with PBS twice before staining. Next, surface staining of anti-CD4-FITC and anti-CD25-PE-Cy7 was performed for 30 min at 4°C. Followed by fixation and permeabilization for 30 min at 4°C, anti-Foxp3-PE or anti-IL-17A-PE antibodies were added and incubated at 4°C for 30 min, respectively. Cells were analyzed by BD FACS Calibur and data were calculated with FlowJo software. All antibodies were purchased from eBioscience, San Diego, CA. CD4 +CD25+ Foxp3 + cells were recognized as Treg cells and CD4 + IL-17 + cells as Th17 cells.

### Western blot analysis

Colons were homogenized with ice-cold strong lysis buffer (Beyotime, China) containing protease inhibitors. The extracts were separated on 8% SDS-PAGE and then transferred to polyvinylidene fluoride membranes. The membrane was blocked with Tris-buffered saline Tween 20 (TBST) buffer containing 5% skim milk and incubated with the following primary antibodies: rabbit anti-mouse Foxp3 (1:250 dilution, Abcam); rabbit anti-mouse RORγt (1:500 dilution, Abcam) and rabbit anti-mouse β-actin (1:1000 dilution, Abcam). The samples were incubated overnight followed by the addition of horseradish peroxidase (HRP) conjugated anti-rabbit IgG secondary antibodies (1:5000 dilution, Santa Cruz Biotechnology). The signals were visualized *via* enhanced chemiluminescence (ECL) detection system (Millipore) according to the manufacturer’s protocol. The protein levels were quantified using Image J software and expressed as the ratio to β-actin.

### ELISA for murine cytokines

Serum was collected from murine blood and prepared for ELISA analysis as previously (Guan et al., 2021). Splenocyte suspensions were prepared from all mice and single cells (5×10^6^ cells/well) were cultured in RPMI-1640 with 10% FCS and 1% penicillin/streptomycin(Sigma-Aldrich). The cultures were then incubated at 37 °C in 5% CO_2_ stimulated with SEA (2 µg/ml) for 72 h. The concentrations of TGF-β1, IL-10, and IL-17A in mouse serum or splenocyte supernatant were determined with ELISA kits (R&D Systems) according to the manufacturer’s instructions.

### Quantitative real-time polymerase chain reaction PCR (qPCR)

Total RNA was collected with a TRIzol^®^ Reagent kit (Invitrogen, Thermo Fisher Scientific, USA) from the pulverized colon or cultured T cells according to the manufacturer’s instruction. And cDNA was produced *via* reverse transcription with a High-Capacity cDNA Reverse Transcription kit (Applied Biosystems, Thermo Fisher Scientific). qPCR was performed using Power Syber Green PCR Master Mix (Applied Biosystems, Foster City, CA, USA) with gene-specific primers as listed in **Table 1**. The amplification reactions were carried out with an initial hold step (95°C for 5 minutes), followed by 36 cycles of a three-step PCR (94°C for 30 seconds, 68°C for 40 seconds, and 72°C for 30 seconds), and a final extension at 72°C for 5 minutes. Quantification of target gene expression was evaluated in the terms of the comparative cycling threshold (Ct) normalized by glyceraldehyde 3-phosphate genase (GAPDH) with the 2^−ΔΔCt^ method.

**Table 1:** Primer sequences of target mRNA.

Genes	Forward (5′ → 3′)	Reverse (5′ → 3′)
*Foxp3* *TGF-β1* *IL-10* *RORγt* *IL-17* *c-MYC* *HIF-1α* *PK* *PKM2* *PFK* *GLUT1* *GLUT4* *PPARa* *CPT1* *MCAD* *L-FABP* *FAS* *ACC* *SCD1* *CD36* *CS* *IDH3G* *GAPDH*	CCCATCCCCAGGAGTCTTG GGGCTGATCCCGTTGAT GCTCTTACTGACTGGCATGAG GACCCACACCTCACAAATTGA TTTAACTCCCTTGGCGCAAAA TTGAAGGCTGGATTTCCTTTGGGC GTCGGACAGCCTCACCAAACAG CAGCCATGGCTGACACCTTC GGAGCCACTCTGAAGATCACC GCCACTAAGATGGGTGCTAAGG GCAGTTCGGCTATAACACTGG GATTCTGCTGCCCTTCTGTC CTGTCGGGATGTCACACAATGC GTGTCCAAGTATCTGGCAGT TAACATACTCGTCACCCTTC TTGACGACTGCCTTGACT TCGGAGACAATTCACCAAACC CACCAGTTTTGCATTGAGAA CTTCCTCCTGAATACATCCCTCC TGGTCAAGCCAGCTAGAAA CGAATTTGAAAGATGTACTGAGC GAGTGGTGACCCGGCAC GTGTTTCCTCGTCCCGTAG	ACCATGACTAGGGGCACTGTA CCCACTGATACGCCTGAG CGCAGCTCTAGGAGCATGTG AGTAGGCCACATTACACTGCT CTTTCCCTCCGCATTGACAC TCGTCGCAGATGAAATAGGGCTGT TAGGTAGTGAGCCACCAGTGTCC GGATCAGATGCAAAGCTTTCTG ACTTCTCCATGTAAGCGTTGTCC CGTACTTGGCTAGGATTTTGAGG GCGGTGGTTCCATGTTTGATTG ATTGGACGCTCTCTCTCCAA TCTTTCAGGTCGTGTTCACAGGTAA CAGGGTATTTCTCAAAGTCAAC ATGCCTGTGATTCTTGCT GCCAGGAGAACTTTGAGC AGCCATCCCACAGGAGAAACC TACGCTGTTGAGTTCATAGGCC CTCCATCCCATCTAGCACAACCT TCCCAAGTAAGGCCATCTC CTTAGGCAGCATTTTCTGGC TCCATCACCCAGTTTCATGATG ATGGCAACAATCTCCACTTT

### Statistical analysis

All experimental data were presented as means ± SEMs and analyzed with SPSS v19.0 (SPSS, Chicago, IL, USA). All *in vivo* studies were repeated two independent times, while the *in vitro* experiments were repeated three times. The significance between the two groups was identified using Student’s t-test. Comparisons among multiple groups were performed by one-way multi-variate analysis of variance (ANOVA) and followed by LSD *post-hoc* test for comparison between every two groups. Degree of significance of *P* < 0.05 was considered as significant while *P* < 0.01 as extremely significant. Graphs were generated with the software GraphPad Prism v.7.0.

## Results

### Pre-exposure to *S. japonicum* eggs alleviates the colon inflammation in the TNBS-induced experimental colitis

**Figure 1:**
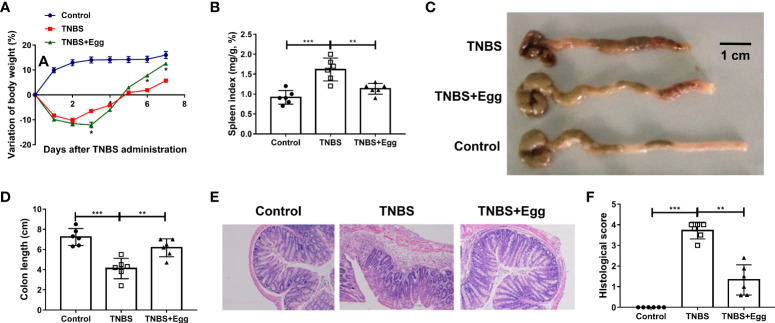
Pre-exposure to *S. japonicum* eggs alleviated the colon inflammation in the TNBS-induced experimental colitis. Female BALB/c mice were randomly divided into 3 groups: Control, TNBS, and TNBS+Egg. TNBS (0.5mg per mouse in 50% ethanol) was used by rectal instillation to induce colitis. Mice of the TNBS+Egg group were pre-exposed to *S. japonicum* eggs as a preventive intervention. **(A)** Dynamics of body weight variation at different time points. *P < 0.01, the TNBS group versus the TNBS+Egg group. All samples including B to F were collected on the 5th day after the TNBS modeling. **(B)** Spleen index. **(C)** Representative images of colons and **(D)** the statistical graphs of colon length. **(E)** Representative images of colon histopathology and **(F)** the statistical graphs of the colonic histological score. Data are expressed as means ± SEMs based on 6 mice in each group and from 2 independent experiments. Asterisks mark significant differences among different groups (**P < 0.01, ***P < 0.001).

Consistent with the previous studies, TNBS instillation in mice led to reduced activities, decreased appetite, piloerection, and bloody stool. To determine the effects of *S. japonicum* eggs on the experimental colitis, we compared parameters of weight loss, colon length, spleen index, and intestinal inflammation between all groups. Murine body weights initially decreased and bottomed out on the 3^rd^ day after modeling in both the TNBS and TNBS+Egg groups, and then gradually improved to the baseline level in the TNBS+Egg group while still lower than those of the control in the TNBS group (**Figure 1A**). In addition, compared with the TNBS group, the TNBS+Egg group lost more weight on the 3^rd^ day after modeling, but accelerated weight gain from the 5^th^ day after modeling. Therefore, the 5^th^ day after modeling was chosen for the following experiment. Spleen enlargement and colon shortening are common characteristic symptoms of colitis (Wirtz et al., 2017). As shown in **Figures 1B–D**, the TNBS group had spleen enlargement and significant colon shortening compared with the control. The egg treatment resulted in positive trends for reversing spleen enlargement and colon shortening in the murine model. Moreover, TNBS induced histological abnormalities and inflammatory cell infiltration, while the egg-treated mice presented mild colonic damage and reduced infiltration of inflammatory cells (**Figures 1E, F**). Taken together, pre-exposure to *S. japonicum* eggs alleviates damaging inflammation in the colon of mice with experimental colitis.

### Pre-exposure to *S. japonicum* eggs upregulates Treg immune response in the TNBS-induced experimental colitis

**Figure 2:**
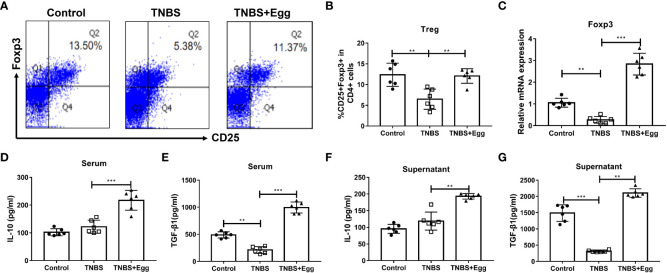
Pre-exposure to *S. japonicum* eggs upregulated Treg immune response in the TNBS-induced experimental colitis. **(A, B)** The proportion of CD25+Foxp3+Treg in CD4+ T cells in spleen. **(C)** The mRNA expression level of Foxp3. The concentrations of IL-10 and TGF-β1 in serum (**D, E**, respectively) and in splenic supernatants (**F, G**, respectively). Data are expressed as means ± SEMs based on 6 mice in each group and from 2 independent experiments. Asterisks mark significant differences among different groups (**P < 0.01, ***P < 0.001).

Previous studies have highlighted the critical role of Treg responses in immune homeostasis, with defects in the number and suppressive function of Treg cells in patients with Crohn’s disease (Clough et al, 2020). To confirm whether *S. japonicum* eggs could amplify Treg response in the TNBS-induced colitis, we compared the frequencies, transcription factor, and functional cytokines of splenic Treg among different groups on the 5^th^ day after the TNBS model. As **Figures 2A–C** revealed, the TNBS group had lower Treg frequency related to the control, accompanied by inhibited mRNA expression of spleen Foxp3, while all the decreases were remarkably reversed by the administration of eggs. To verify the effects of eggs on Treg function in the TNBS mice, we next detected expression levels of the functional cytokines IL-10 and TGF-β1 in serum and splenocyte supernatant. The results (**Figures 2D–G**) showed that the TNBS group had lower TGF-β1 concentration than the control, however, concentrations of IL-10 and TGF-β1 were significantly elevated with pre-exposure of *S. japonicum* eggs in the colitis mice. Collectively, the above data imply that pre-exposure to *S. japonicum* eggs upregulates Treg immune response in the murine experimental colitis.

### Pre-exposure to *S. japonicum* eggs downregulates Th17 immune response in the TNBS-induced experimental colitis

**Figure 3:**
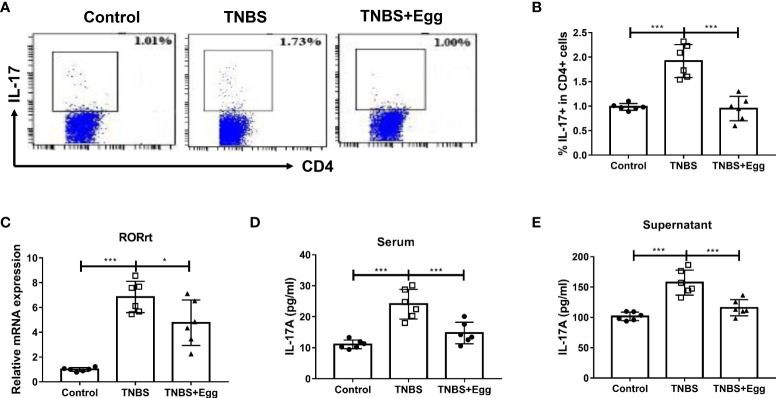
Pre-exposure to *S. japonicum* eggs downregulated Th17 immune response in the TNBS-induced experimental colitis. The proportion of IL-17+ Th17 in spleen CD4+ T cells **(A, B)**. The mRNA expression level of Th17 specific transcription factor RORγt in spleen **(C)**. The concentration of IL-17A in serum **(D)** and in splenic supernatant **(E)**. Data are expressed as means ± SEMs based on 6 mice in each group and from 2 independent experiments. Asterisks mark significant differences among different groups (*P < 0.05, ***P < 0.001).

Th17 responses are linked to the development of inflammatory diseases, which underpin severe morbidity in IBDs (Alexander et al., 2022). To explore the role of egg treatment on Th17 immune responses in murine colitis, we detected the frequencies of Th17 in different groups. Consistent with the previous studies, the TNBS group had increased percentages of splenic Th17 relative to the control (**Figures 3A, B**), accompanied by enhanced mRNA expression of RORγt in the spleen (**Figure 3C**). However, administration of *S. japonicum* eggs inhibited all the above parameters to varying degrees in the colitis mice (**Figures 3A–C**). To verify the effect of egg treatment on Th17 function in the colitis mice, next we detected IL-17A concentration in serum and splenocyte supernatant from all groups. As **Figures 3D, E** indicated, the TNBS group had upregulated concentrations of IL-17A in comparison with the control. However, pre-exposure with eggs restored elevated IL-17A levels caused by the TNBS administration in the mice. Taken together, our results show that pre-exposure to *S. japonicum* eggs downregulates the Th17 population and function in the TNBS-induced experimental colitis.

### The protective effect of *S. japonicum* eggs on the experimental colitis is associated with Treg and Th17 balance in mice

**Figure 4:**
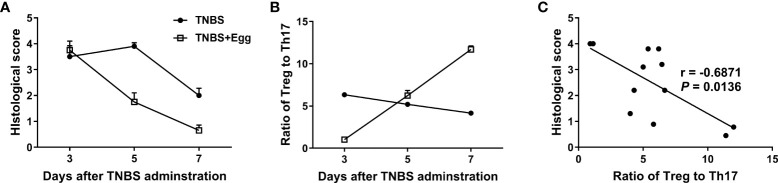
The colon inflammation had a correlation with the Treg/Th17 ratio in the TNBS-induced experimental colitis. Dynamics of histological scores **(A)** and ratio of Treg to Th17 **(B)** in mice with the experimental colitis at the indicated time points. **(C)** Correlation analysis of the histological scores and the ratio of Treg to Th17 in the experimental colitis.

Given that Treg and Th17 imbalance plays a crucial role in inflammatory diseases including IBDs (Yan et al., 2020), we asked whether the shift in splenic Treg/Th17 balance toward Treg response in the mice receiving eggs was associated with the inflammation process in colitis. Thus, we investigated the histological score in colon tissues and the ratio of splenic Treg/Th17 on the 3^rd^, 5^th^, and 7^th^ days post the TNBS modeling. As **Figures 4A, B** shown, the histological score in the TNBS+Egg group were higher than that of the TNBS group on the 3^rd^ day, while much lower than those of the TNBS group on the 5^th^ and 7th days after the TNBS modeling, respectively. However, the ratios of Treg/Th17 had the opposite trend in the groups. Furthermore, the ratio of Treg/Th17 was correlated negatively with the histological scores in the experiment groups (**Figure 4C**). Thus, these results suggest that the protective effect of *S. japonicum* eggs on the murine experimental colitis is associated with Treg/Th17 immune balance.

### Regulation of Treg/Th17 balance by SEA *via* modifying glucose and lipid metabolism *in vitro*

**Figure 5:**
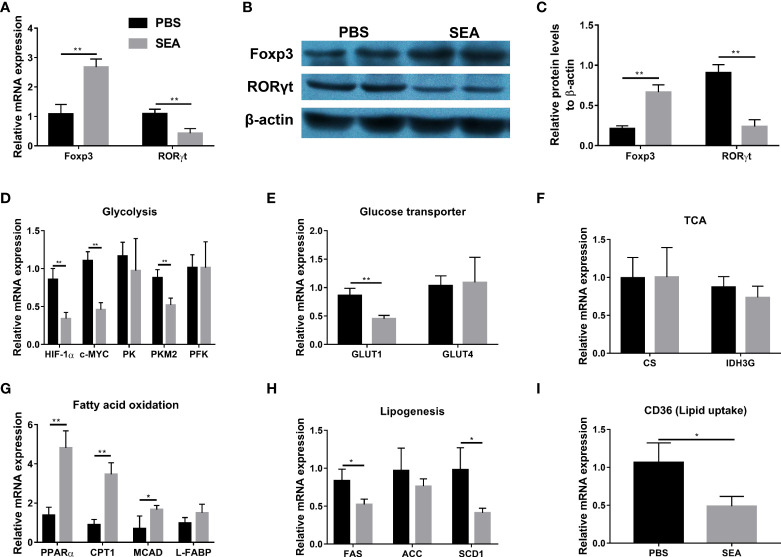
Modification of Treg and Th17 expansion by SEA *via* regulating glucose and lipid metabolism *in vitro*. Spleen naïve CD4+ T cells (CD4+ CD25- CD44- CD62L+) were sorted from the TNBS mice on the 5^th^ day post TNBS administration. T cells were cultured (3×10^5^ cells/well) in 96-well plates coated with anti-CD3 mAb (5 μg/mL) and soluble anti-CD28 mAb (3 μg/mL) and kept in presence of IL-2 (100 U/mL). SEA (10 µg/mL) was added and PBS used as the control. Cells were cultured in RPMI-1640 Medium, 10% fetal calf serum, 2 mM L-glutamine, and 100 U/mL of Penicillin/Streptomycin for 3 days. The relative mRNA expression levels of Foxp3 and RORγt **(A)**. The protein expressions of Foxp3 and RORγt **(B, C)**. The relative mRNA expression levels of genes associated with glycolysis **(D)**, glucose transporter **(E)**, TCA **(F)**, fatty acid oxidation **(G)**, lipogenesis **(H)** and lipid uptake **(I)** were detected. Data are expressed as means ± SEMs based on 3 samples in each group and from 3 independent experiments. Asterisks mark significant differences among different groups (*P< 0.01, **P < 0.01).

It has been demonstrated that *Schistosoma* and *Schistosoma*-derived products have a strong ability to regulate the glucose and lipid metabolism in the host body (Cai et al., 2021). Given metabolism plays an important role in CD4+ cells differentiation (Chapman et al., 2020; Guan et al., 2020; Qin et al., 2022), we hypothesized that *S. japonicum* eggs might regulate Treg/Th17 through modulating metabolism in the murine experimental colitis. To test our hypothesis, we sorted naïve CD4+ T cells from the TNBS group and then co-cultured them with SEA *in vitro*, since SEA is the primary active component secreted by *S. japonicum* eggs (Xu et al., 2019). As shown in **Figures 5A–C**, SEA could promote Treg expansion and inhibit Th17 development, evidenced by increased mRNA and protein expressions of Foxp3 and decreased mRNA and protein expressions of RORγt, as compared with the PBS control. To investigate glucose and lipid metabolism-related genes involved in this process, we detected mRNA expression of related genes in cultured cells. As **Figures 5D–F** indicated, SEA treatment led to the down-regulated expression of glycolysis-related genes (Hypoxia-inducible factor 1α, HIF-1α; c-MYC; Pyruvate kinase isozyme type M2, PKM2), and GLUT1 gene (Glucose transporter 1), but had no effect on other genes (Pyruvate kinase, PK; Phosphofructokinase, PFK; GLUT4) involved in these processes and tricarboxylic acid cycle (TCA) related genes (Citrate synthase, CS; Isocitrate dehydrogenase 3, IDH3G). In addition, the mRNA levels of fatty acid (FA) oxidation-related genes (Peroxisome proliferator-activated receptor alpha, PPARα; Carnitine palmitoyl transferase 1, CTP-1; Medium-chain acyl-CoA Dehydrogenase, MCAD) were significantly upregulated by SEA treatment, while the FA synthesis genes (Fatty acid synthase, FAS; and Stearoyl-CoA desaturase 1, SCD1) and lipid uptake gene (CD36) were downregulated by SEA treatment (**Figures 5G–I**). L-FABP (Liver-fatty acid binding protein) and ACC (Acetyl coenzyme A carboxylase) involved in these processes did not show any change (**Figures 5G, H**). Taken together, these results provide evidence that SEA promotes naïve CD4+ T cells to differentiate into Treg, which was associated with the effect of SEA on inhibiting the glycolysis pathway and promoting the FA oxidation pathway *in vitro*.

### Pre-exposure to *S. japonicum* eggs regulates glucose and lipid metabolism in the colon tissue from the TNBS-induced colitis mice

**Figure 6:**
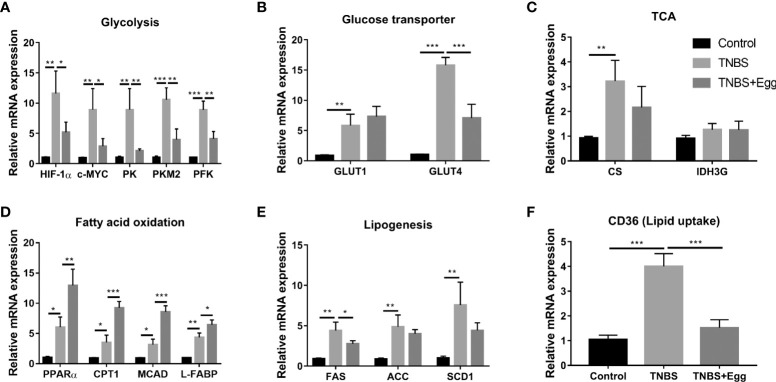
Pre-exposure to *S. japonicum* eggs led to reprogramming of glucose and lipid metabolism in colon tissue from mice with experimental colitis. The relative mRNA expression levels of genes related to glycolysis **(A)**, glycose transporters **(B)**, TCA **(C)**, fatty acid oxidation **(D)**, lipogenesis **(E)**, and lipid uptake **(F)** in colon tissues were evaluated by RT-PCR. Data are expressed as means ± SEMs based on 6 samples in each group and from 2 independent experiments. Asterisks mark significant differences among different groups (*P< 0.01, **P < 0.01, ***P < 0.001).

Recent investigations indicate *S. japonicum* infection leads to the reprogramming of glucose and lipid metabolism in hosts, which plays an important role in cross-talk between helminths and inflammation diseases (Yang et al., 2021; Dai et al., 2022). Therefore, we explored the possible effects of egg treatment on intestinal glucose and lipid metabolism in the colon tissues of all groups. As shown in **Figures 6A–C**, compared with the control, the TNBS group had higher mRNA expressions of glycolytic pathway related genes (HIF-1α; c-MYC; PK; PKM2; PFK); glycose transport related genes (GLUT1 and GLUT4); TCA related genes (CS), although gene expression of IDH3G associated with TCA did not demonstrate any remarkable change. In comparison with the TNBS group, pre-exposure to *S. japonicum* eggs resulted in lower expressions of glycolytic pathway related genes and GLUT4 in the murine colon with the experimental colitis. Besides, the TNBS administration increased mRNA expressions of FA oxidation genes (PPARα; CTP-1; MCAD; L-FABP), lipogenesis-related genes (FAS; ACC; SCD1), and lipid uptake gene (CD36) in the murine colon (**Figures 6D–F**) However, all the mentioned parameters of FA oxidation genes in the colon tissues were upregulated by egg treatment to varying degrees, while the colonic expressions of lipogenesis-related genes and lipid uptake genes were inhibited by egg treatment. Together, these results indicate that there is upregulated glucose and lipid metabolism in the colon tissues of the experimental colitis mice. In consistency with these aforementioned *in vitro* results, pro-exposure with *S. japonicum* egg results in reprogramming of glycolipid metabolism in the colon with TNBS colitis.

## Discussion

This study aimed to explore the underlying mechanism by which *S. japonicum* eggs alleviated the acute colon inflammation in the murine TNBS colitis. The findings presented demonstrate that *S. japonicum* eggs confer a protective effect on colitis and the protective effect is associated with restoring Treg and Th17 balance and reprogramming glycolipid metabolism.

TNBS colitis is a key model of acute colitis form and provides a murine model that resembles human Crohn’s disease to explore the immune responses in IBDs (Wirtz et al., 2007; Wirtz et al., 2017). Since this model is associated with an increase in highly activated T cells and as such T cells play an essential role in TNBS colitis, it is suitable for ascertaining CD4+T cell-dependent immunity in the inflammatory bowel (Wirtz et al., 2017). During active colitis, intestinal inflammation is typically associated with diarrhea and reduced food intake, and thus determining the murine body weight is usually important in estimating the severity of colitis. Differences in body weight curves between animal groups are generally indicative of changes in colitis activity between experimental groups (Wirtz et al., 2017). In this study, we observed that TNBS administration rapidly led to severe colitis with the typical signs of dramatic body weight loss on the 1^st^ day and unrecoverable original weight until the 7^th^ day, accompanied by the lowest body weight on the 3^rd^ day. These results are in accordance with previous studies demonstrated by Aharoni et al. (Aharoni et al., 2005) and Hollenbach et al. (Hollenbach et al., 2005).

It is well established that helminths can protect mice from experimental colitis (Ruyssers et al., 2009; Shi et al., 2022). However, in the present study, pre-exposure to eggs aggravated body weight loss in the mice with colitis on the 3^rd^ day post TNBS inoculation, although it had no effect on the mortality of mice (data not shown). It might be associated with transient damage caused by *S. japonicum* eggs, because a previous study reports that orthotopic freeze-dried *S. japonicum* eggs result in granulomatous inflammation in the cerebral tissue of rabbits (Xu et al., 2013). From the 5^th^ day after modeling, *S. japonicum* egg treatment significantly abrogated overall disease manifestations and inflammatory parameters. That is the reason why this study chose the 5^th^ day after the TNBS administration as the main checkpoint. Collectively, in line with the previous studies (Elliott et al., 2003; Mo et al., 2007; Mu et al., 2021; Shi et al., 2022), egg pretreatment has a protective effect on the experimental colitis in mice.

How eggs modulate colitis has been an area of intense research by several groups, including ours. The Treg-Th17 axis plays an important role in the regulation of intestinal immune responses, and the loss of the balance between regulatory and effector T cells is thought to lead to the development of IBDs (Ueno et al., 2018; Yan et al., 2020). Given the important role of the Treg-Th17 axis in the progression of IBDs, we asked whether the protective effect of *S. japonicum* eggs on the experimental colitis was associated with their regulation of Treg and Th17 immune responses. The data indicated that TNBS administration led to inhibited frequencies and function of Treg and increased frequencies and function of Th17, which are consistent with the previous report (Lim et al., 2016). The colon inflammation caused by TNBS is most likely due to the downregulation of Treg and upregulation of Th17 differentiation during the colitis. However, relative to the TNBS mice, Treg frequency and function were upregulated, while Th17 frequency and function were downregulated in the TNBS+Egg mice. Furthermore, the ratio of Treg/Th17 was correlated negatively with the histological scores in the experiment groups. Studies have shown that Treg cells prevent the occurrence of autoimmune diseases critically depending on the effect factors TGF-β and IL-10, while Th17 cells promote autoimmune and inflammatory processes mainly by secreting IL-17A closely involved with the severity of IBDs (Britton et al., 2019; Yan et al., 2020). Our data support that the corrected Treg/Th17 immune balance is associated with the protective role of *S. japonicum* eggs in murine colitis. Further studies will be required to explore the detailed molecular mechanism of how eggs regulate Treg/Th17 responses in TNBS colitis. Collectively, this study reveals that the beneficial role of S*. japonicum* eggs in promoting resistance to TNBS-induced damage is related to restoring the Treg/Th17 balance.

It has become increasingly clear that T cell activation, differentiation, and effector functions are closely associated with cellular metabolic programs, including glycolysis, FA synthesis, and mitochondrial metabolism (Bantug et al., 2018). There is compelling evidence that the etiology of IBDs may be associated with various metabolic dysregulations (Alexander et al., 2022; Zheng et al., 2022). It has been demonstrated that parasite-derived products have a strong ability to regulate metabolism in the host body (Zhu et al., 2014; Cai et al., 2021; Fisher et al., 2021). Differentiation of T cells can be manipulated through modulating metabolic activity *in vitro* (Chang et al., 2013). Therefore, we hypothesized that the regulation of *S. japonicum* eggs on the Treg-Th17 axis might be associated with glycolipid metabolism in experimental colitis.

The *in vitro* experiment results showed that stimulation of SEA, the main active constituent of eggs, could promote naïve CD4+ T cells to differentiate in favor of Treg, and decreased gene expressions related to glycolysis (HIF-1α, c-MYC, and PKM2), accompanied by increased gene expressions related to FA oxidation. Murine studies have shown that HIF-1α promotes Th17 but inhibits Treg cell differentiation when naive T cells are activated (Bettelli et al., 2006). And HIF-1α deficiency results in greatly reduced glycolytic activity, and thus impairment in the Th17 differentiation, but promoted the development of Treg (Shi et al., 2011). Therefore, the HIF-1α-dependent glycolysis pathway is critical for regulating Th17 and Treg differentiation. In addition, Myc is another critical regulator in T cell metabolism as a transcription factor for GLUT1 (the main glucose transporter in lymphocytes) and PK involved in glycolysis (Gnanaprakasam et al., 2017). It is reported that the PKM2 inhibitor suppresses glycolysis and Th17 differentiation by inhibiting PK activity, thereby alleviating disease activity in experimental autoimmune encephalomyelitis (Kono et al., 2019). Therefore, downregulation of the transcription factor c-Myc is critical in inhibiting glycolysis (Qin et al., 2022). Collectively, decreased glycolytic pathways could shift immune cells from a pro-inflammatory state to anti-inflammatory status.

Numerous studies have proved that lipid metabolism is important in Treg and Th17 balance, among which Treg cells rely mainly on FA oxidation whereas Th17 cells are dependent on FA synthesis (Berod et al., 2014; Wang et al., 2015). Our data indicated that SEA not only facilitated FA oxidation (PPARα, CPT1, and MCAD), but also inhibited lipid synthesis (FAS, ACC, and SCD1) and lipid uptake (CD36) in T cells during the *in vitro* study. Such metabolic changes promoted differentiation of Treg and inhibit the development of Th17. Several studies have shown that both *S. japonicum* infection and stimulation with SEA *in vitro* significantly enhance the mRNA levels of FA oxidation-related genes, but decrease genes related to FA synthesis (FAS, ACC, and SCD1) and lipid uptake (CD36) in macrophages (Xu et al., 2019; Guan et al., 2020; Cai et al., 2021). Our results were consistent with the previous reports. A Murine study has demonstrated that differentiation of Treg is inhibited following treatment with an inhibitor of CPT1 (Michalek et al., 2011). ACC is the key enzyme of *de novo* fatty acid synthesis. Th17 cell differentiation depends on ACC1-mediated *de novo* FA synthesis, and ACC1 deletion suppresses Th17 immune response in murine models of colitis (Mamareli et al., 2021). It is reported that glycolysis alone is not sufficient to support Th17 cell differentiation without the help of *de novo* FA synthesis (Berod et al., 2014; Mamareli et al., 2021). Therefore, inhibition of SEA on Th17 differentiation is associated with both decreased glycolysis and FA synthesis in the present study. Compared to effector CD4+ T cells, Treg cells depend on FA oxidation to fuel mitochondrial respiration (Shi et al., 2011). Interestingly, SEA did not affect TCA-related gene expressions (CS and IDH3G) in the *in vitro* experiment, and further study is needed to clarify it. Taken together, modulation of SEA on the lipid metabolism affected the differentiation of CD4+ T cells, and inhibition of FA synthesis or promotion of FA oxidation modulates the Th17/Treg axis in favor of Treg cells.

It has been proved that the ultimate effect of intracellular metabolism on immune cell functions is not only influenced by the balance of the different metabolic pathways, but also by external stimuli (Chapman et al., 2020). A previous study reveals that *S. japonicum* infection induces the reprogramming of glucose and lipid metabolism in the murine colon (Yang et al., 2021). Our data demonstrated that the TNBS+Egg group had lower expressions of glycolytic pathway related genes and GLUT4 genes in the colon tissue than the TNBS group. In addition, the gene expressions involved in the FA oxidation were upregulated by egg treatment, while the FA synthesis gene related to lipogenesis was decreased in the colon with TNBS colitis. These findings are in keeping with the results from the *in vitro* experiment, which enables an assessment of the importance of parasite eggs in treating colitis by regulating glycolipid metabolism.

A large number of studies, including ours, have confirmed that eggs induce the differentiation of naive T cells into Treg cells, while the underlying complex mechanism is still elusive. It has been reported that SEA can protect against type 1 autoimmune diabetes *via* increasing Foxp3 expression in a TGF-β-dependent manner, along with upregulating C-type lectins, IL-10, and IL-2 on dendritic cells (DC). These effects of SEA on T cells and DC may act in synergy to induce Foxp3+ Treg in NOD mice (Zaccone et al., 2009). In addition, inflammatory cytokines released from damaged tissues caused by eggs, such as IL-10 and IL-33, can induce Treg differentiation (He et al., 2018; Bai et al., 2021). The components of *S. japonicum* egg antigens are very complex. Assessing which components of SEA have immunomodulatory effects on Treg will be a prerequisite to exploring helminth-derived molecules as novel immune agents. The major antigenic glycoprotein ω-1 in SEA has been identified to induce Foxp3 expression in naive T cells dependent on TGF-β and retinoic acid (Zaccone et al., 2011). Another group has reported that heat shock protein 60 (SjHSP60), derived from *S. japonicum* eggs, drives both *de novo* induction and direct expansion of preexisting Tregs in a TLR4-dependent manner (Zhou et al., 2018). Although our present data indicate the induction of Treg by eggs or SEA is associated with regulating glucose and lipid metabolism, further studies are needed to explore the active component of SEA and its underlying molecular mechanism of Treg induction by eggs in experimental colitis induced by TNBS.

In summary, the present study provides novel insights on the protective role of *S. japonicum* eggs and the underlying mechanism in treating TNBS colitis, focusing on Treg and Th17 balance and reprogramming glycolipid metabolism. These findings provide experimental evidence for the further exploration of *S. japonicum* eggs as a potential candidate in the treatment of IBDs. More in-depth investigations are essential to explore the detailed molecular mechanism by which eggs remodel glycolipid metabolism and then regulate the Treg and Th17 immune balance.

## Data availability statement

The raw data supporting the conclusions of this article will be made available by the authors, without undue reservation.

## Ethics statement

The animal study was reviewed and approved by Institutional Animal Care and Utilization Committee of Tongji Medical College, Huazhong University of Science and Technology, China (SCXK2020-0018).

## Author contributions

Conceived and designed: JL. Performed the experiments: XH, FZ, WZ, and FG. Analyzed the data: XH, WZ, MJ, and JL. Contributed to materials-related issues: XH, JH, and JL. Drafted the manuscript: XH and JL. Supervised the study: JH, FG, and JL. All authors contributed to the article and approved the submitted version.

## Funding

This work was funded by grants from the National Nature Science Foundation of China (81772220) and the Fundamental Research Funds for the Central Universities (HUST 2016YXMS199).

## Conflict of interest

The authors declare that the research was conducted in the absence of any commercial or financial relationships that could be construed as a potential conflict of interest.

## Publisher’s note

All claims expressed in this article are solely those of the authors and do not necessarily represent those of their affiliated organizations, or those of the publisher, the editors and the reviewers. Any product that may be evaluated in this article, or claim that may be made by its manufacturer, is not guaranteed or endorsed by the publisher.

## References

[B1] Aden K. Rehman A. Waschina S. Pan W. H. Walker A. Lucio M.. (2019). Metabolic functions of gut microbes associate with efficacy of tumor necrosis factor antagonists in patients with inflammatory bowel diseases. Gastroenterology 157, 1279–1292.e11. doi: 10.1053/j.gastro.2019.07.025 31326413

[B2] Aharoni R. Kayhan B. Arnon R. (2005). Therapeutic effect of the immunomodulator glatiramer acetate on trinitrobenzene sulfonic acid-induced experimental colitis. Inflamm. Bowel. Dis. 11, 106–115.35. doi: 10.1097/00054725-200502000-00003 15677903

[B3] Alexander M. Ang Q. Y. Nayak R. R. Bustion A. E. Sandy M. Zhang B.. (2022). Human gut bacterial metabolism drives Th17 activation and colitis. Cell Host Microbe 30, 17–30.e9. doi: 10.1016/j.chom.2021.11.001 34822777 PMC8785648

[B4] Bai Y. Guan F. Zhu F. Jiang C. Xu X. Zheng F.. (2021). IL-33/ST2 axis deficiency exacerbates hepatic pathology by regulating treg and Th17 cells in murine schistosomiasis japonica. J. Inflamm. Res. 14, 5981–5998. doi: 10.2147/JIR.S336404 34815688 PMC8604654

[B5] Bantug G. R. Galluzzi L. Kroemer G. Hess C. (2018). The spectrum of T cell metabolism in health and disease. Nat. Rev. Immunol. 18, 19–34. doi: 10.1038/nri.2017.99 28944771

[B6] Berod L. Friedrich C. Nandan A. Freitag J. Hagemann S. Harmrolfs K.. (2014). *De novo* fatty acid synthesis controls the fate between regulatory T and T helper 17 cells. Nat. Med. 20, 1327–1333. doi: 10.1038/nm.3704 25282359

[B7] Bettelli E. Carrier Y. Gao W. Korn T. Strom T. B. Oukka M.. (2006). Reciprocal developmental pathways for the generation of pathogenic effector TH17 and regulatory T cells. Nature 441, 235–238. doi: 10.1038/nature04753 16648838

[B8] Britton G. J. Contijoch E. J. Mogno I. Vennaro O. H. Llewellyn S. R. Ng R.. (2019). Microbiotas from humans with inflammatory bowel disease alter the balance of gut Th17 and RORγt+ regulatory T cells and exacerbate colitis in mice. Immunity 50, 212–224.e4. doi: 10.1016/j.immuni.2018.12.015 30650377 PMC6512335

[B9] Cai Z. Deng X. Zhao L. Wang X. Yang L. Yuan G. (2021). The relationship between *Schistosoma* and glycolipid metabolism. Microb. Pathog. 159, 105120. doi: 10.1016/j.micpath.2021.105120 34358648

[B10] Chang J. T. (2020). Pathophysiology of inflammatory bowel diseases. N. Engl. J. Med. 383, 2652–2664. doi: 10.1056/NEJMra2002697 33382932

[B11] Chang C. H. Curtis J. D. Maggi L. B. Jr. Faubert B. Villarino A. V. O’Sullivan D.. (2013). Posttranscriptional control of T cell effector function by aerobic glycolysis. Cell 153, 1239–1251. doi: 10.1016/j.cell.2013.05.016 23746840 PMC3804311

[B12] Chapman N. M. Boothby M. R. Chi H. (2020). Metabolic coordination of T cell quiescence and activation. Nat. Rev. Immunol. 20, 55–70. doi: 10.1038/s41577-019-0203-y 31406325

[B13] Chen J. Y. Zhou J. K. Pan W. (2021). Immunometabolism: Towards a better understanding the mechanism of parasitic infection and immunity. Front. Immunol. 12, 661241. doi: 10.3389/fimmu.2021.661241 34122419 PMC8191844

[B14] Clough J. N. Omer O. S. Tasker S. Lord G. M. Irving P. M. (2020). Regulatory T-cell therapy in crohn’s disease: challenges and advances. Gut 69, 942–952. doi: 10.1136/gutjnl-2019-319850 31980447 PMC7229901

[B15] Dai M. Yang X. Yu Y. Pan W. (2022). Helminth and host crosstalk: New insight into treatment of obesity and its associated metabolic syndromes. Front. Immunol. 13, 827486. doi: 10.3389/fimmu.2022.827486 35281054 PMC8913526

[B16] Elliott D. E. Li J. Blum A. Qadir K. Urban J. F. Jr. Weinstock J. V. (2003). Exposure to schistosome eggs protects mice from TNBS-induced colitis. Am. J. Physiol. Gastroint. Liver. Physiol. 284, G385–G391. doi: 10.1152/ajpgi.00049.2002 12431903

[B17] Fisher J. R. Chroust Z. D. Onyoni F. Soong L. (2021). Pattern recognition receptors in innate immunity to obligate intracellular bacteria. Zoonoses 1, 10. doi: 10.15212/ZOONOSES-2021-0011 35282331 PMC8909792

[B18] Gnanaprakasam J. N. R. Sherman J. W. Wang R. (2017). MYC and HIF in shaping immune response and immune metabolism. Cytokine Growth Factor. Rev. 35, 63–70. doi: 10.1016/j.cytogfr.2017.03.004 28363691

[B19] Guan F. Jiang W. Bai Y. Hou X. Jiang C. Zhang C.. (2021). Purinergic P2X7 receptor mediates the elimination of *Trichinella spiralis* by activating NF-κB/NLRP3/IL-1β pathway in macrophages. Infect. Immun. 89, e00683–e00620. doi: 10.1128/IAI.00683-20 33558327 PMC8091101

[B20] Guan F. Zhang C. Jiang C. Jacques M. L. Bai Y. Lu S.. (2020). ApoE deficiency promotes hepatic pathology by aggravating Th17/Treg imbalance in murine schistosomiasis japonica. Parasite. Immunol. 42, e12785. doi: 10.1111/pim.12785 32786078

[B21] He L. Zhou S. Qi Q. Chi Y. Zhu J. Xu Z.. (2018). The regulation of regulation: interleukin-10 increases CD4+ CD25+ regulatory T cells but impairs their immunosuppressive activity in murine models with schistosomiasis japonica or asthma. Immunology. 153, 84–96. doi: 10.1111/imm.12813 28799262 PMC5721254

[B22] Hodson R. (2016). Inflammatory bowel disease. Nature 540, S97. doi: 10.1038/540S97a 28002398

[B23] Hollenbach E. Vieth M. Roessner A. Neumann M. Malfertheiner P. Naumann M. (2005). Inhibition of RICK/nuclear factor-kappaB and p38 signaling attenuates the inflammatory response in a murine model of crohn disease. J. Biol. Chem. 280, 14981–14988. doi: 10.1074/jbc.M500966200 15691843

[B24] Kono M. Maeda K. Stocton-Gavanescu I. Pan W. Umeda M. Katsuyama E.. (2019). Pyruvate kinase M2 is requisite for Th1 and Th17 differentiation. JCI Insight 4, e127395. doi: 10.1172/jci.insight.127395 PMC6629104 31217348

[B25] Lamb C. A. Saifuddin A. Powell N. Rieder F. (2022). The future of precision medicine to predict outcomes and control tissue remodeling in inflammatory bowel disease. Gastroenterology 162, 1525–1542. doi: 10.1053/j.gastro.2021.09.077 34995532 PMC8983496

[B26] Lim S. M. Jeong J. J. Choi H. S. Chang H. B. Kim D. H. (2016). Mangiferin corrects the imbalance of Th17/Treg cells in mice with TNBS-induced colitis. Int. Immunophar. 34, 220–228. doi: 10.1016/j.intimp.2016.03.004 26971225

[B27] Mak W. Y. Zhao M. Ng S. C. Burisch J. (2020). The epidemiology of inflammatory bowel disease: East meets west. J. Gastroenterol. Hepatol. 35, 380–389. doi: 10.1111/jgh.14872 31596960

[B28] Mamareli P. Kruse F. Lu C. W. Guderian M. Floess S. Rox K.. (2021). Targeting cellular fatty acid synthesis limits T helper and innate lymphoid cell function during intestinal inflammation and infection. Mucosal Immunol. 14, 164–176. doi: 10.1038/s41385-020-0285-7 32355319

[B29] Michalek R. D. Gerriets V. A. Jacobs S. R. Macintyre A. N. MacIver N. J. Mason E. F.. (2011). Cutting edge: distinct glycolytic and lipid oxidative metabolic programs are essential for effector and regulatory CD4+ T cell subsets. J. Immunol. 186, 3299–3303. doi: 10.4049/jimmunol.1003613 21317389 PMC3198034

[B30] Mo H. M. Liu W. Q. Lei J. H. Cheng Y. L. Wang C. Z. Li Y. L. (2007). *Schistosoma japonicum* eggs modulate the activity of CD4+ CD25+ tregs and prevent development of colitis in mice. Exp. Parasitol. 116, 385–389. doi: 10.1016/j.exppara.2007.02.009 17433300

[B31] Mu Y. McManus D. P. Hou N. Cai P. (2021). Schistosome infection and schistosome-derived products as modulators for the prevention and alleviation of immunological disorders. Front. Immunol. 12, 619776. doi: 10.3389/fimmu.2021.619776 33692793 PMC7937812

[B32] Na S. Y. Moon W. (2019). Perspectives on current and novel treatments for inflammatory bowel disease. Gut. Liver. 13, 604–616. doi: 10.5009/gnl19019 31195433 PMC6860034

[B33] Neurath M. F. (2014). Cytokines in inflammatory bowel disease. Nat. Rev. Immunol. 14, 329–342. doi: 10.1038/nri3661 24751956

[B34] Neurath M. F. Fuss I. Kelsall B. L. Stuber E. Strober W. (1995). Antibodies to interleukin 12 abrogate established experimental colitis in mice. J. Exp. Med. 182, 1281–1290. doi: 10.1084/jem.182.5.1281 7595199 PMC2192205

[B35] Noack M. Miossec P. (2014). Th17 and regulatory T cell balance in autoimmune and inflammatory diseases. Autoimmun. Rev. 13, 668–677. doi: 10.1016/j.autrev.2013.12.004 24418308

[B36] Qin Y. Gao C. Luo J. (2022). Metabolism characteristics of Th17 and regulatory T cells in autoimmune diseases. Front. Immunol. 13, 828191. doi: 10.3389/fimmu.2022.828191 35281063 PMC8913504

[B37] Ruyssers N. E. De Winter B. Y. De Man J. G. Loukas A. Pearson M. S. Weinstock J. V.. (2009). Therapeutic potential of helminth soluble proteins in TNBS-induced colitis in mice. Inflamm. Bowel. Dis. 15, 491–500. doi: 10.1002/ibd.20787 19023900

[B38] Savage N. (2016). Q&A: Joel weinstock. Nature 540, S103. doi: 10.1038/540S103a 28002396

[B39] Shi L. Z. Wang R. Huang G. Vogel P. Neale G. Green D. R.. (2011). HIF1alpha-dependent glycolytic pathway orchestrates a metabolic checkpoint for the differentiation of TH17 and treg cells. J. Exp. Med. 208, 1367–1376. doi: 10.1084/jem.20110278 21708926 PMC3135370

[B40] Shi W. Xu N. Wang X. Vallée I. Liu M. Liu X. (2022). Helminth therapy for immune-mediated inflammatory diseases: Current and future perspectives. J. Inflamm. Res. 15, 475–491. doi: 10.2147/JIR.S348079 35087284 PMC8789313

[B41] Ueno A. Jeffery L. Kobayashi T. Hibi T. Ghosh S. Jijon H. (2018). Th17 plasticity and its relevance to inflammatory bowel disease. J. Autoimmun. 87, 38–49. doi: 10.1016/j.jaut.2017.12.004 29290521

[B42] Wang S. Huang J. Tan K. S. Deng L. Liu F. Tan W. (2022). Isosteviol sodium ameliorates dextran sodium sulfate-induced chronic colitis through the regulation of metabolic profiling, macrophage polarization, and NF-κB pathway. Oxid. Med. Cell. Longev. 2022, 4636618. doi: 10.1155/2022/4636618 35126813 PMC8813272

[B43] Wang C. Yosef N. Gaublomme J. Wu C. Lee Y. Clish C. B.. (2015). CD5L/AIM regulates lipid biosynthesis and restrains Th17 cell pathogenicity. Cell 163, 1413–1427. doi: 10.1016/j.cell.2015.10.068 26607793 PMC4671820

[B44] Wirtz S. Neufert C. Weigmann B. Neurath M. F. (2007). Chemically induced mouse models of intestinal inflammation. Nat. Protoc. 2, 541–546. doi: 10.1038/nprot.2007.41 17406617

[B45] Wirtz S. Popp V. Kindermann M. Gerlach K. Weigmann B. Fichtner-Feigl S.. (2017). Chemically induced mouse models of acute and chronic intestinal inflammation. Nat. Protoc. 12, 1295–1309. doi: 10.1038/nprot.2017.044 28569761

[B46] Xu Z. P. Chang H. Ni Y. Y. Li C. Chen L. Hou M.. (2019) *Schistosoma japonicum* infection causes a reprogramming of glycolipid metabolism in the liver. Parasite. Vectors 12, 388. doi: 10.1186/s13071-019-3621-6 PMC6679454 31375125

[B47] Xu J. Lu X. J. Wang D. Wu M. C. Chen S. J. Li J. C.. (2013). Preliminary study on establishing an animal model of neuroschistosomiasis by direct injection of *Schistosoma japonicum* eggs through skull. Zhongguo. Xue. Xi. Chong. Bing. Fang. Zhi. Za. Zhi. 25, 28–30. doi: 10.16250/j.32.1374.2013.01.015 23687807

[B48] Yang X. Ding W. Qian X. Jiang P. Chen Q. Zhang X.. (2021). *Schistosoma japonicum* infection leads to the reprogramming of glucose and lipid metabolism in the colon of mice. Front. Vet. Sci. 8, 645807. doi: 10.3389/fvets.2021.645807 33791356 PMC8006365

[B49] Yan J. B. Luo M. M. Chen Z. Y. He B. H. (2020). The function and role of the Th17/Treg cell balance in inflammatory bowel disease. J. Immunol. Res. 2020, 8813558. doi: 10.1155/2020/8813558 33381606 PMC7755495

[B50] Zaccone P. Burton O. T. Gibbs S. E. Miller N. Jones F. M. Schramm G.. (2011). The *S. mansoni* glycoprotein ω-1 induces Foxp3 expression in NOD mouse CD4^+^ T cells. Eur. J. Immunol. 41, 2709–2718. doi: 10.1002/eji.201141429 21710488

[B51] Zaccone P. Burton O. Miller N. Jones F. M. Dunne D. W. Cooke A. (2009). *Schistosoma mansoni* egg antigens induce treg that participate in diabetes prevention in NOD mice. Eur. J. Immunol. 39, 1098–1107. doi: 10.1002/eji.200838871 19291704

[B52] Zhao Y. Zhang S. Jiang L. Jiang J. Liu H. (2009). Preventive effects of *Schistosoma japonicum* ova on trinitrobenzenesulfonic acid-induced colitis and bacterial translocation in mice. J. Gastroenterol. Hepatol. 24, 1775–1780. doi: 10.1111/j.1440-1746.2009.05986.x 20136961

[B53] Zheng L. Wen X. L. Duan S. L. (2022). Role of metabolites derived from gut microbiota in inflammatory bowel disease. World J. Clin. cases 10, 2660–2677. doi: 10.12998/wjcc.v10.i9.2660 35434116 PMC8968818

[B54] Zhou S. Qi Q. Wang X. Zhang L. Xu L. Dong L.. (2018). SjHSP60 induces CD4+ CD25+ Foxp3+ tregs *via* TLR4-mal-drived production of TGF-β in macrophages. Immunol. Cell Biol. 96, 958–968. doi: 10.1111/imcb.12160 29697865 PMC6197892

[B55] Zhu J. Xu Z. Chen X. Zhou S. Zhang W. Chi Y.. (2014). Parasitic antigens alter macrophage polarization during *Schistosoma japonicum* infection in mice. Parasite. Vectors 7, 122. doi: 10.1186/1756-3305-7-122 PMC3975460 24666892

